# Flavor characteristics of garlic fish cakes using electronic nose and tongue analyses

**DOI:** 10.1038/s41598-024-55341-w

**Published:** 2024-03-13

**Authors:** Hae-Yeon Choi, Hye-Eun Woo, Eun-Seong Go, Jin-Seong Kim, Jin-Hee Choi

**Affiliations:** 1 Department of Food Service Management and Nutrition, Kongju National University, Yesan-gun, Chungcheongnam-do 32439 Republic of Korea; 2 Department of Food Science and Nutrition, Daejin University, Pocheon-si, Gyeonggi-do 11159 Republic of Korea

**Keywords:** Chemical physics, Screening

## Abstract

This study investigated the utility of garlic powder as a functional ingredient. The aim was to develop fish cakes with improved functionality and sensory preference based on the antioxidant activity and quality characteristics. Increasing amounts of garlic powder in the prepared fish cakes were associated with increasing total polyphenol and flavonoid contents, 2,2-diphenyl-1-picrylhydrazyl (DPPH) and 2,2′-azino-bis(3-ethylbenzothiazolin-6-sulfonic acid) (ABTS^+^) radical scavenging activity, and reducing power. Furthermore, electronic tongue and electronic nose analyses showed an increased the intensity of umami and sourness and increased the levels of volatile compounds. The lowest trimethylamine peak corresponded to the highest amount of garlic powder. Sensory evaluation indicated that 3% garlic powder had the highest score for all criteria. Fishy odor decreased as the proportion of garlic powder increased. These findings suggest that the addition of 3% garlic powder improves quality characteristics, sensory preference, and antioxidant activity of fish cakes.

## Introduction

Fish cakes are produced through a variety of processes, from boiling, steaming, frying, and grilling of a dough containing a mixture of fish paste, which is prepared by adding salt to fish fillet with subsequent elution of salt-soluble proteins, and additional ingredients, such as flour, starch, and seasoning, among others. The type of fish cake product thus varies according to the manufacturing method^1,2^. Product diversity is high because of the wide range of fish and additional ingredients that can be incorporated. Compared to other animal protein-based products, fish cakes are relatively low in cost and are thus a popular food choice^3^. The market has recently undergone changes in product differentiation and enhancement toward ready-to-eat bakery type fish cakes. The current trend is an increased demand for fish cakes containing functional materials consistent with consumer preference and health-oriented consumption patterns, with various studies ongoing^3,4^. The most notable studies have reported the addition of functional materials such as lotus leaf powder^5^, turmeric powder^6^, yam powder^7^, mealworm larval powder^8^, propolis^9^, and aronia extract^10^. Nevertheless, in most previous studies, fish cakes were prepared by frying; despite the hygienic quality and low risk of microbial contamination associated with this high-temperature cooking process, the potential drawbacks include high calorie loads and lipid acidification^10,11^. Therefore, recent research on fish cakes lead to the development of baked and steamed forms. For this study, the fish cake in this study will be manufactured by baking.

Fishy odor is another important problem in the production of various fish-based processed foods. Postmortem decaying of fish and shellfish promotes the production of volatile nitrogen compounds responsible for fishy odor and other malodors, such as putrefactive smell. Trimethylamine, the main cause of the fishy odor, is generated through the reduction of trimethylamine-N-oxide, mediated by microorganisms and enzymes, which affects product quality^12^. In the past, condiment plants, such as leek, ginger, garlic, onion, and radish, were used to remove undesirable smells. In western cooking, spices and herbs, such as onion, celery, parsley, thyme, and whole pepper, were used for a similar purpose^13^. Studies investigating the odor-masking effects of plant ingredients have reported that *Allium sativum* L. (garlic, Amaryllidaceae family) decreases the intensity of fishy odor^14,15^.

Garlic is a perennial herb originating from central Asia^16^. In Korean cooking, garlic has been used as a medicinal herb, as well as a spice for removing unwanted odor and stimulating appetite^17^. Garlic contains *S*-allyl-l-cysteine sulfoxide, diallyl thiosulfate, ajoene, and scordinine, which exhibit antioxidant, antimicrobial, immune-enhancing, anticancer, antiviral, antithrombotic, and hypoglycemic effects^18,19^. Notably, *S*-allyl-l-cysteine sulfoxide, also known as alliin, has been shown to improve blood cholesterols, which led to its acceptance as a notified functional material by the Ministry of Food and Drug Safety in South Korea^20^. Previous studies on the development of high-quality processed foods using garlic have mainly focused on cookies^21^, grilled pork patties^17^, Tteokbokki or simmered rice cake^22^ and raw noodle^23^. However, few studies have used garlic as a functional material and characterized the flavor qualities of the resulting products.

This study investigated the antioxidant activities and quality of fish cakes prepared using varying amounts of garlic powder. Specifically, we assessed the flavor and taste profiles using electronic nose and tongue analyses and performed sensory evaluation to identify processed fish products with enhanced functionality and consumer preference.

## Materials and methods

### Test materials

Garlic powder was purchased from HeungSeong Food Co. Ltd. (Hongcheon, Korea). Fish fillet was provided from DongKwang Food Co. (Busan, Korea). Salt (CJ CheilJedang Co., Sinan, Korea), Sugar (CJ CheilJedang Co., Incheon, Korea), Wheat Flour (Daehan Flour Co., Incheon, Korea), Cooking oil (Sajodaerim Co., Incheon, Korea) were purchased from a local supermarket. The experimental reagents 2,2-diphenyl-1-picrylhydrazyl, 2 N Folin & Ciocalteu reagent, gallic acid, quercetin, and 2,2′-azino-bis(3-ethylbenzothiazoline-6-sulfonic acid) were purchased from Sigma–Aldrich Co. (St. Louis, MO, USA). Ethyl alcohol, sodium carbonate, sodium hydroxide, and potassium persulfate, among others, were purchased from Daejung Chemicals & Metals Co. (Siheung, Korea). All reagents and solvents were of Grade 1 quality.

### Preparation of baked fish cakes with added garlic powder

Grilled fish cakes with added garlic powder were prepared in a pilot study as previously described^24^. The mixing ratio is shown in Table 1. The fish fillet was loosened in a blender (BL642KR, SharkNinja LLC, Needham, MA, USA) for 1 min. Then salt and sugar were added and blended for 1 min, garlic powder was added at 0%, 1%, 2%, 3%, or 4% of the batter, wheat flour was added in the other proportions, and mixed it. The resulting dough was divided into small portions of equal weights on an oven pan (2 × 4 × 8.5 cm^3^) using a piping bag. The dough was steamed at 100 °C for 10 min, then grilled at 150 °C for another 10 min in a convection oven (Libero 240914, Electrolux, Stockholm, Sweden). The baked fish cakes were cooled at 25 °C for 10 min prior to analysis.

**Table 1:** Formula for fish cakes added with different amounts of garlic powder.

Ingredient (g)	CON	1%	2%	3%	4%
Fish fillet	277.2				
Salt	3.6				
Sugar	7.2				
Wheat flour	40.0	36.4	32.8	29.2	25.6
Garlic powder	–	3.6	7.2	10.8	14.4
Cooking oil	7.2				
Total	360				

CON: fish cake with 0% garlic powder added.

1%: fish cake with 1% garlic powder added.

2%: fish cake with 2% garlic powder added.

3%: fish cake with 3% garlic powder added.

4%: fish cake with 4% garlic powder added.

### Preparation of sample solution

The sample solution was prepared using 10 g of garlic powder mixed with 90 mL of 70% ethyl alcohol, extracted in a shaker (SI-900R, Jeio Tech, Gimpo, Korea) at 20 °C for 24 h, and filtered. The fish cake extracted by ground 10 g with 90 mL of 70% ethyl alcohol following the same process as the powder.

### Total polyphenol content

Total polyphenol content was measured as Folin-Ciocalteu method^25^. To 100 μL of sample solution, 200 μL of 2 N Folin-Ciocalteu reagent and 2 mL of distilled water were added, and the mixture was shaken and allowed to stand in the dark for 3 min. Next, 2 mL of 10% sodium carbonate (Na_2_CO_3_) was added and allowed to react in dark for 1 h. The resulting solution was measured at 765 nm using a spectrophotometer (DU-800, Beckman coulter, Inc., Brea, CA, USA). Standard curve was plotted using gallic acid as the reference, and total polyphenol content was expressed as mg gallic acid equivalents per 100 g sample (mg GAE/100 g).

### Total flavonoid content

Total flavonoid content was measured as described who applied the Davis method with modifications^26^. To 100 μL of sample solution, 1 mL of 90% diethylene glycol and 100 μL of 1 N NaOH were added, and the mixture was shaken and allowed to stand in a water bath (SB-1200, Eyela, Tokyo, Japan) at 37 °C for 1 h. The absorbance was measured at 420 nm using the spectrophotometer. Standard curve was plotted using quercetin as the reference, and total flavonoid content was expressed as mg quercetin equivalents per 100 g sample (mg QE/100 g).

### DPPH radical scavenging activity

DPPH radical scavenging activity was measured as described by prior literature^27^. To 4 mL of sample solution, 1 mL of 0.15 mM DPPH solution was added. The mixture was allowed to stand in dark for 30 min, and the absorbance was measured at 517 nm using the spectrophotometer. The absorbance was measured in the control group by adding ethanol to the sample solution. The scavenging activity was calculated using the following equation: DPPH radical scavenging activity (%) = [(1−sample group absorbance)/control group absorbance] × 100.

### ABTS^+^ radical scavenging activity

ABTS^+^ radical scavenging activity was measured as described by prior literature^28^. To a 7.0 mM ABTS^+^ solution, 2.45 mM potassium persulfate was added, and the mixture allowed to react for 24 h in dark. The ABTS^+^ solution with radicals was diluted using ethyl alcohol until the absorbance, measured at 734 nm using the spectrophotometer, reached 0.70 ± 0.02. After mixing 100 μL of sample solution and 900 μL of ABTS^+^ solution, absorbance values were obtained over 6 min at 1 min intervals. The absorbance was measured in the control group by adding ethanol to the sample solution. The scavenging activity was calculated in a similar manner as for DPPH radical scavenging activity.

### Reducing power

Reducing power was measured as described by prior literature^29^. To 2.5 mL of sample solution, 2.5 mL of 1% potassium ferricyanide and 2.5 mL of 0.2 M sodium phosphate buffer (pH 6.6) were added, and the mixture was allowed to react in a water bath at 50 °C for 20 min. To the resulting solution, 2.5 mL of 10% trichloroacetic acid was added, and the mixture was centrifuged at 1935 × *g* after 10 min (Sorvall legend RT, Thermo Fisher Scientific Inc., Waltham, MA, USA). Thereafter, 2.5 mL of the supernatant was mixed with 2.5 mL of distilled water. The resulting solution was mixed with 1 mL of 0.1% ferric chloride, and the absorbance was measured at 700 nm using the spectrophotometer to express the reducing power.

### pH

To measure the pH, 5 g of garlic powder and fish cake were each mixed with 45 mL of distilled water and blended for 30 s (BL642KR, SharkNinja LLC, Needham, MA, USA). The solution was filtered, and measurements were taken using a pH meter (FEP-20, Mettler Toledo, Burrington, UK).

### Water content

To measure the water content, 0.5 g of sample was taken from the center of the fish cake, and measurements were taken by an infrared moisture analyzer (MJ-33, Mettler Toledo, Zurich, Switzerland).

### Color

To measure the color, the L (lightness), a (+ redness/– greenness), and b (+ yellowness/– blueness) values were obtained using a colorimeter (CR-400, Konica Minolta Co., Osaka, Japan). The standard plate corresponded to L = 94.66, a = − 0.42, and b = 4.11.

### Textural properties

To measure the mechanical texture, the samples were prepared in 1.5 × 1.5 × 1.5 cm^3^ samples, and a two-bite test was performed using a texture analyzer (TA-XT2, Stable Micro System, Ltd., Haslemere, UK). A plunger of 75 mm∮ was used to measure the hardness, springiness, adhesiveness, chewiness, cohesiveness, and gumminess at a test speed of 1.0 mm/s, strain of 70%, pre-test speed of 2.0 mm/s, and post-test speed of 5.0 mm/s.

### Electronic tongue analysis

A 20 g sample was mixed with 80 mL distilled water for 1 min in a homogenizer (Polytron PT-2500 E, Kinematica AG, Malters, Switzerland). The solution was filtered and the supernatant was extracted, diluted, and placed in a glass container. For the electronic tongue analysis (Astree V, Alpha MOS, Toulouse, France), 0.1 M HCl, 0.1 M NaCl, and 0.1 M MSG were used as the references to measure the sourness, saltiness, and umami, respectively, and draw the taste profile. The taste sensors were as follows: NMS (umami), CTS (saltiness), AHS (sourness), with PKS, ANS, SCS and CPS sensors used as standards. The taste profile was obtained as a radar chart using AlphaSoft (Alpha MOS, Toulouse, France).

### Electronic nose analysis

For an electronic nose analysis, 5 g of sample was placed in a vial, and an electronic nose (Heracles II, Alpha MOS, Toulouse, France) was applied, whereby measurements were taken after saturating the volatile compounds under the following conditions: headspace temperature, 60 °C; flow rate, 250 mL/min; quantity injection, 2.5 mL; and acquisition time, 120 s. The data were analyzed and obtained as a chromatogram using Alphasoft (Alpha MOS, Toulouse, France).

### Sensory evaluation

The sensory evaluation involved a panel of 25 trained raters, who were informed of the study purpose and items to be tested prior to evaluation, as well as a detailed description of each assessment characteristic. After being cut into uniform sizes (1.5 × 1.5 × 3 cm^3^) and cooled at room temperature for 20 min, the samples were provided in a white polyethylene plate labeled with a 3-digit number from a random number table. The items of consumer preference (appearance, flavor, taste, texture, and overall preference) were rated on a 9-point scale (1 = highly unfavorable, 9 = highly favorable). The items of quality (fishy odor, chewiness, and garlic flavor) were also rated on a 9-point scale (1 = very weak, 9 = very strong). For the sensory evaluation experiment, we obtained informed consent to participate in the experiment from all subjects. And this study was approved by the Institutional Review Board of Kongju National University and conducted according to the relevant regulations (Approval Number: KNU_IRB_2022-44).

### Statistical analysis

Statistical analysis was performed using SPSS 25.0 (IBM SPSS Inc., Armonk, NY, USA). Data are expressed as the mean and standard deviation. One-way ANOVA was performed to analyze differences between samples with Duncan’s multiple range test, and significance was set at P < 0.05.

## Results and discussion

### Antioxidant activities of fish cakes with added garlic powder

#### Total polyphenol and flavonoid contents

**Figure 1:**
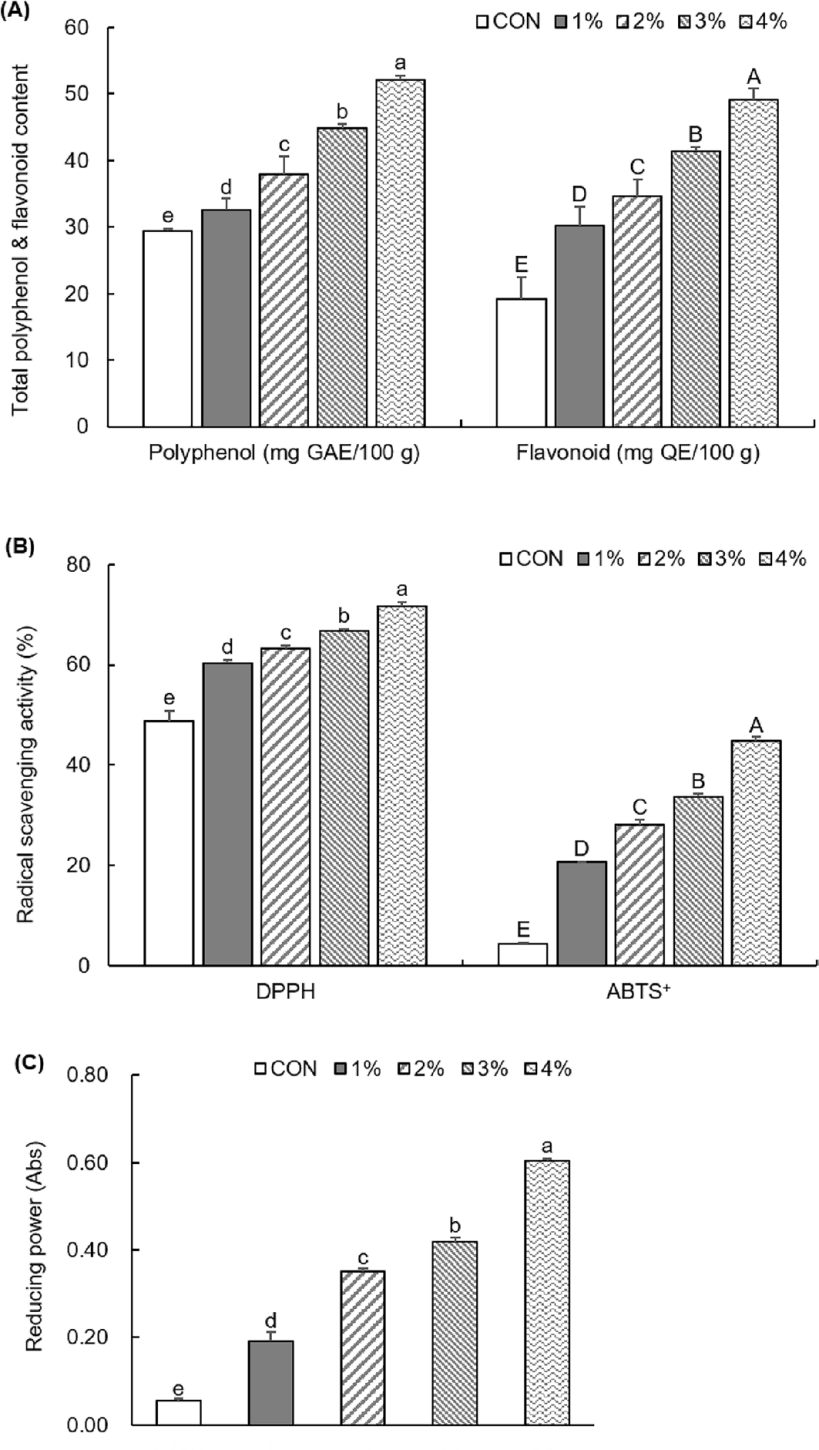
(**A**) Total polyphenol and flavonoid contents of baked fish cakes added with different amounts of garlic powder. (**B**) DPPH and ABTS^+^ radical scavenging activities of baked fish cakes added with different amounts of garlic powder. (**C**) Reducing power of baked fish cakes added with different amounts of garlic powder. The different letters (a–e, A–E) are meaning significant difference at the figure (P < 0.05).

Figure 1A presented the total polyphenol and flavonoid contents of fish cakes. The total polyphenol and flavonoid contents of the garlic powder were 4.01 mg GAE/g and 3.18 mg QE/g, respectively. The total polyphenol content of the fish cakes was 29.38 mg GAE/100 g in the control group and 32.57–52.15 mg GAE/100 g in the garlic-powder groups. The total flavonoid content of the fish cakes was 19.11 mg QE/100 g in the control group and 30.23–49.11 mg QE/100 g in the garlic-powder groups. These results indicated that the total polyphenol and flavonoid contents increased with increasing amounts of garlic powder (both P < 0.05). Reported that the phenolic content in Yanggaeng (sweet jelly) increased as the amount of added garlic extract increased^30^. Phenolic compounds are widely distributed in natural flora, and their varied amounts and molecular structures of phenolic hydroxyl groups enables their binding to proteins and other macromolecules^31^. Flavonoids are the largest group of phyto polyphenols and exhibit various bioactivities associated with antioxidant effects^32^. A study investigating pork patties showed that the flavonoid content increased when flavonoid-containing onion extract was used, consistent with our results^33^. Because phenolic compounds, such as flavonoids and phenolic acids, are critical factors in antioxidant activity, we hypothesized that the addition of garlic powder affected the antioxidant activity in the fish cakes by increasing the total polyphenol and flavonoid contents^34^.

#### DPPH–ABTS^+^ radical scavenging activity

Figure 1B presented the DPPH and ABTS^+^ radical scavenging activity of fish cakes with added garlic powder. The DPPH radical scavenging activity of garlic powder at 1 mg/mL was 41.11%. The DPPH radical scavenging activity was 48.85% in the control group and 60.23–71.77% in the garlic-powder groups, which demonstrated an increasing trend with increasing amounts of garlic powder (*p* < 0.001). Studies of cookie, who used garlic and black garlic, showed comparable results in their investigations^35,36^ respectively. The ABTS^+^ radical scavenging activity of garlic powder at 1 mg/mL was 9.19%, and the scavenging activity of the fish cakes was 4.25% in the control group and 20.62%–44.92% in the garlic-powder groups, which demonstrated an increasing trend with increasing amounts of garlic powder (P < 0.001), whereas similar results in their investigations of salted fish sauce and sweet jelly, respectively^30,37^. The extraction solvent and types and properties of the extracted phenolic compounds are known to affect radical scavenging activity, which is the ability of phenolic compounds to remove radicals, with high phenolic contents being associated with stronger radical scavenging effects^38^. These findings suggest that the addition of garlic powder in fish cakes promotes radical scavenging activity by increasing the phenolic contents.

#### Reducing power of garlic powder and fish cakes

Figure 1C presented the reducing power of fish cakes with added garlic powder. The reducing power of garlic powder was 0.25 at 1 mg/mL. The reducing power of fish cakes was 0.06 in the control group and 0.19–0.61 in the garlic-powder groups, which demonstrated an increasing trend with increasing amounts of garlic powder (P < 0.001). Reducing power is an indicator of the ability of an antioxidant to donate electrons to reactive oxygen species or free radicals, and it is measured according to the level of green coloration upon the reduction of Fe^3+^ to Fe^2+^ by the antioxidant^39^. The main bioactive compounds in garlic include sulfur-containing compounds such as diallyl thiosulfate, diallyl disulfide, diallyl trisulfide, and S-allyl-cysteine and phenolic compounds such as β-resorcylic acid, gallic acid, pyrogallol, and quercetin^19^. It has been reported that the addition of garlic to red pepper paste increased the reducing power^40^. In addition, cooking garlic has been shown to promote antioxidant production via a non-enzymatic browning reaction^41^. Hence, the bioactive compounds of the garlic powder is expected to exert a positive functional effect by increasing the reducing power in fish cakes.

### Quality characteristics of fish cakes with added garlic powder

#### pH, water content, and color

Table 2 presented the pH, water content and color of the fish cakes. The pH of the garlic powder was 6.11. The pH of the fish cakes was 7.09 in the control group and 6.87–7.04 in the garlic-powder groups, demonstrating a decreasing trend with increasing amounts of garlic powder (P < 0.001). Prior research has reported a decrease in the pH of steamed fish cakes and sausages, respectively, with increasing amounts of garlic powder^42,43^. Garlic is known to contain various organic acids, including lactic acid, pyruvic acid, malic acid, and citric acid^16^. Hence, increasing the added amount of garlic powder increases the organic acid content and lowers the pH.

**Table 2:** pH, water content and color of fish cakes added with different amounts of garlic powder.

		CON	1%	2%	3%	4%	*F-value*
pH		7.09 ± 0.05^a^	7.04 ± 0.01^b^	6.99 ± 0.02^c^	6.93 ± 0.03^d^	6.87 ± 0.03^e^	47.148***
Water content (%)		51.43 ± 1.57	51.54 ± 0.77	51.58 ± 1.17	51.74 ± 0.53	51.16 ± 0.90	0.171^NS^
Color	L*	74.38 ± 1.56^a^	72.15 ± 1.34^b^	70.61 ± 2.33^c^	69.09 ± 2.31^d^	66.48 ± 2.77^e^	75.476***
	a*	− 0.96 ± 0.55^a^	− 0.13 ± 0.60^b^	0.95 ± 0.84^c^	1.52 ± 0.66^d^	2.63 ± 1.07^e^	127.134***
	b*	11.86 ± 1.19^a^	14.15 ± 1.72^b^	16.33 ± 2.06^c^	17.22 ± 1.63^d^	18.24 ± 1.77^e^	86.338***

All values are mean ± standard deviation (SD) (n ≥ 3).

Different letters within the same row (a–e) differ significantly by Duncan’s multiple range test (P < 0.05).

^NS^Not significant.

***P < 0.001.

The water content of the fish cakes was 51.43% in the control group and 51.16–51.74% in the garlic-powder groups, which showed no significant variation between groups. Previous studies reported that the addition of turmeric powder^6^ and yam powder^7^ had no effect on the water content of fish cakes. These are suggested that a trace amount of additional ingredients had a negligible effect on water content^44^. The lack of substantial variation in this study is attributable to the fact that the amount of garlic powder was insufficient to affect the water content of the fish cakes.

The color test results indicated an L value of 74.38 in the control group and 66.48–72.15 in the garlic-powder groups, demonstrating a reduction in lightness as the amount of garlic powder increased (P < 0.001). The a value was − 0.96 in the control group and − 0.13 to 2.63 in the garlic-powder groups, demonstrating an increase in redness as the amount of garlic powder increased (P < 0.001). The b value was 11.86 in the control group and 14.15–17.22 in the garlic-powder groups, demonstrating increasing yellowness as the amount of garlic powder increased (P < 0.001). It showed comparable results in their investigation of cookies, where the change in color was reported to be associated with the unique light-yellow hue of garlic powder, which is produced by browning via an amino-carbonyl reaction during the high-temperature drying process in garlic powder production^23,45^. These findings suggest that the color of the garlic powder had an effect on the color of the fish cakes in this study.

#### Textural properties

Table 3 presented the textural properties of the fish cakes. Hardness was 2399.54 g in the control group and 1900.69–2315.31 g in the garlic-powder group, indicating that hardness decreased as the amount of garlic powder increased (P < 0.001). Similarly, the hardness of fish cakes decreased as the amount of lees powder increased, which was due to the formation of gluten in the flour upon addition of lees powder and reduced binding between proteins^46^. Increasing amounts of *Undaria pinnatifida* sporophyll (UPS) powder have also been found to reduce the hardness of fish cakes^47^. Another study reported that the hardness of raw noodles decreased as the amount of garlic powder increased, whereas the reduction was attributed to reduced binding between gluten proteins^23^. Therefore, the addition of garlic powder in this study is thought to suppress gluten formation in the flour of the fish cake dough as well as binding between proteins to reduce the hardness of the fish cakes.

**Table 3:** Texture analysis of fish cakes added with different amounts of garlic powder.

	CON	1%	2%	3%	4%	*F-value*
Hardness	2399.54 ± 174.49^a^	2315.31 ± 372.27^ab^	2238.57 ± 257.03^ab^	2161.03 ± 263.26^b^	1900.69 ± 322.93^c^	10.255***
Adhesiveness	− 43.92 ± 9.22^a^	− 46.27 ± 12.72^ab^	− 49.45 ± 5.30^ab^	− 52.00 ± 6.25^bc^	− 57.15 ± 11.98^c^	6.022***
Springiness	0.61 ± 0.05^a^	0.60 ± 0.05^a^	0.58 ± 0.02^a^	0.58 ± 0.04^a^	0.51 ± 0.08^b^	11.056***
Cohesiveness	0.35 ± 0.03^a^	0.33 ± 0.02^ab^	0.33 ± 0.02^b^	0.32 ± 0.02^bc^	0.31 ± 0.02^c^	9.156***
Gumminess	828.53 ± 99.16^a^	770.72 ± 140.70^ab^	731.63 ± 97.29^bc^	691.99 ± 99.81^c^	551.03 ± 86.02^d^	23.912***
Chewiness	505.90 ± 74.80^a^	464.66 ± 99.24^ab^	425.45 ± 64.28^bc^	397.87 ± 53.88^c^	284.52 ± 75.29^d^	29.360***

All values are mean ± standard deviation (SD) (n = 20).

Different letters within the same row (a–d) differ significantly by Duncan’s multiple range test (P < 0.05).

***P < 0.001.

Springiness was 0.51–0.61 in the garlic-powder groups, and although no significant difference was detected up to the addition of 3% garlic powder, the 4% garlic-powder group demonstrated a significant reduction in springiness (P < 0.001). The factors influencing springiness in the production of fish cakes are known to vary according to the type and amount of additional ingredients^8^. The springiness of fish cakes was influenced by pH and additional ingredients, with pH 6.5–7.0 reported to producing the highest degree of springiness^48^. Another study reported that the springiness of fish cakes with added angelica root powder decreased when the amount of additional ingredients exceeded certain levels^49^.

Adhesiveness and cohesiveness were − 57.15 to − 43.92 and 0.31–0.35, respectively. As the added amount of garlic powder increased, adhesiveness and cohesiveness tended to increase and decrease, respectively (P < 0.001). Gumminess and chewiness were 551.03–828.53 and 284.52–505.90, respectively, demonstrating a decreasing trend as the amount of garlic powder increased (P < 0.001). UPS powder was shown to significantly reduce the cohesiveness, gumminess, and chewiness of fish cakes^47^. Additionally, increasing the amounts of lees powder reduced the gumminess and chewiness of fish cakes^46^. These results suggest that the textural properties of fish cakes are influenced considerably by the additional ingredients and characteristics of these ingredients. Notably, when hardness decreased because of the addition of garlic powder, springiness was maintained up to a certain level, indicating that the addition of an adequate amount of garlic powder would maintain a soft texture and suitable springiness and thus benefit the textural quality of fish cakes.

#### Electronic tongue results

**Figure 2:**
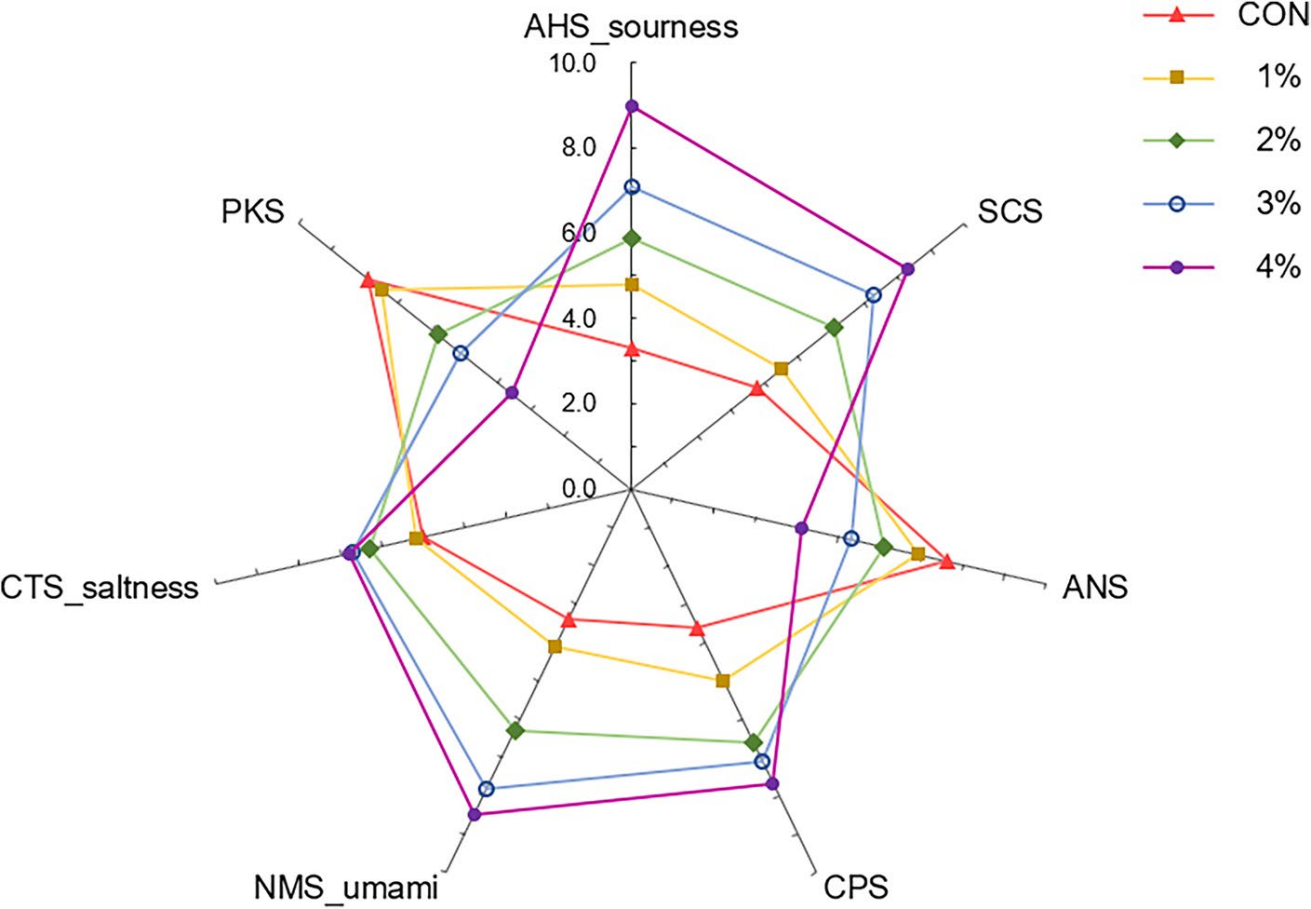
Radar chart for taste attributes of fish cakes added with different amounts of garlic powder.

Figure 2 presented the radar chart based on the scores of the converted AHS, CTS, and NMS sensor results. We observed a score of 3.3 for sourness (AHS) in the control group and 4.8, 5.9, 7.1, and 9.0 in the groups with 1%, 2%, 3%, and 4% garlic powder, respectively; 5.0 for saltiness (CTS) in the control group and 5.2, 6.3, 6.7, and 6.8 in the groups with 1%, 2%, 3%, and 4% garlic powder, respectively; 3.4 for umami (NMS) in the control group and 4.1, 6.3, 7.8, and 8.5 in the groups with 1%, 2%, 3%, and 4% garlic powder, respectively. These results all demonstrated an increased reactivity across all tested items as the amount of garlic powder increased. According to the manufacturer of the electronic nose, the difference in reactivity should be ≥ 2.0 for a human rater to perceive the difference in taste intensity^50^. Despite the increased saltiness in this study, the difference was < 2.0 in both the control and garlic-powder groups, which would be imperceptible to humans. In contrast, the increase in sourness is likely to be perceivable because the difference from that in the control group was apparent from the addition of 2% garlic powder. Reported that sourness increases with increasing free fatty acid content as a result of triglyceride degradation by allicin^51^. Sourness could also be influenced by the organic acids in garlic^16^. The increase in umami is expected to be perceivable because there was a notable difference from that in the control group with the addition of 2% garlic powder. Reported that umami increased because of various sulfur compounds in garlic powder^17^. Garlic has also been reported to contain free amino acids that contribute to umami, such as glutamic acid and aspartic acid^21^. These results suggest that the addition of increasing amounts of garlic powder would cause a corresponding increase in the contents of sulfur compounds and free amino acids, thereby enhancing umami and promoting the sensory quality of fish cakes.

#### Electronic nose results

**Figure 3:**
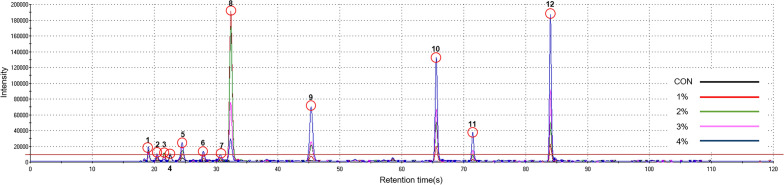
Chromatogram for volatile compounds of fish cakes added with different amounts of garlic powder. 1, Methyl formate; 2, trimethylamine; 3, trimethylamine; 4, acetaldehyde; 5, butane-2,3-dione; 6, ethyl acetate; 7, allyl mercaptan; 8, butanal; 9, 2,3-pentanedione; 10, 2,3-dimethylpyrazine; 11, tetramethylpyrazine; 12, ethenyl-dimethylpyrazine.

Figure 3 presented the result of the electronic nose analysis with varying amounts of garlic powder. As the amount of garlic powder increased, the contents of methyl formate, acetaldehyde, butane-2,3-dione, ethyl acetate, allyl mercaptan, butanal, 2,3-pentanedione, 2,3-dimethylpyrazine, tetramethylpyrazine, and ethenyl-dimethylpyrazine increased (corresponding to peaks 1, 5, 6, 7, 9, 10, 11, and 12, respectively). Acetaldehyde and butanal at peaks 4 and 8 are low-molecular compounds with high volatility found naturally in fish fillets^52^. Butane-2,3-dione, ethyl acetate, allyl mercaptan, and 2,3-pentanedione (peaks numbered 5, 6, 7, and 9, respectively) are responsible for the flavor of garlic; allyl mercaptan, in particular, is produced during heat treatment and is a known metabolite of allium derivatives that inhibit cholesterol synthesis^53^. 2,3-Dimethylpyrazine, tetramethylpyrazine, and ethenyl-dimethylpyrazine are heterocyclic pyrazines, which are produced upon non-enzymatic browning of garlic, such as through the Maillard reaction^54,55^. These compounds are presumed to have been produced through non-enzymatic browning reactions of reduced sugars and amino acids during high-temperature drying in the production of the garlic powder, and oven-baking of the fish cakes could have generated such flavor compounds. Peaks 2 and 3 indicate trimethylamine, the cause of fishy odor^56^. The lowest intensity for these peaks were observed in the 4% garlic-powder group, as the strong flavor of sulfur compounds in garlic masked the fishy odor^14^. These findings suggest that the addition of garlic powder in fish cakes would enhance the overall flavor and simultaneously reduce fishy odor.

#### Sensory evaluation results

Table 4 presented the sensory evaluation results with varying amounts of garlic powder. Fish cakes with 3% garlic powder scored the highest. Regarding quality intensity, with the increase in the amount of garlic powder, the fishy odor decreased, whereas the flavor of garlic increased. We hypothesized that the fishy odor was reduced by alk(en)yl-l-cysteine sulfoxide to enhance consumer preference^17^. Fish cake taste is associated with enhanced flavor, and we found that umami increased with increasing amounts of garlic powder. The taste and flavor scores were high presumably due to the increase in volatile aromatic compounds. However, the addition of ≥ 4% garlic powder negatively affected preference likely due to the increase in the unique garlic flavor and taste. Similarly, for the case of cookie, preference decreased when the intensity of garlic flavor and taste increased to an excessive level^57^. It was reported that springiness was a quality determinant of fish cakes^3^; the textural preference in this study was associated with the 4% garlic-powder group, though this groups showed significantly reduced springiness in the textural measurements. Thus, the addition of 3% garlic powder to fish cakes would enhance preference and reduce fishy odor and may represent the optimal amount to produce fish cakes with outstanding qualities and functionality.

**Table 4:** Sensory characteristics of fish cakes added with different amounts of garlic powder.

		CON	1%	2%	3%	4%	*F-value*
Acceptability	Appearance	4.44 ± 1.95^bc^	4.61 ± 2.45^bc^	5.78 ± 2.21^ab^	6.89 ± 2.30^a^	4.17 ± 1.58^c^	5.174**
	Flavor	3.50 ± 1.42^c^	3.83 ± 1.69^c^	5.83 ± 1.34^ab^	6.72 ± 1.84^a^	5.22 ± 2.16^b^	11.187***
	Taste	4.50 ± 2.20^c^	4.17 ± 1.98^bc^	5.83 ± 1.79^ab^	6.56 ± 2.23^a^	3.67 ± 2.09^c^	5.010**
	Texture	4.94 ± 2.21^bc^	4.67 ± 2.14^bc^	5.89 ± 1.60^ab^	6.39 ± 1.58^a^	3.72 ± 2.44^c^	4.818**
	Overall preference	4.06 ± 2.51^c^	4.39 ± 1.46^bc^	5.50 ± 2.01^ab^	6.78 ± 1.31^a^	4.17 ± 2.33^bc^	6.160***
Intensity	Fishy odor	6.11 ± 2.70^a^	5.78 ± 2.02^ab^	4.94 ± 1.83^abc^	4.44 ± 1.85^bc^	3.78 ± 2.16^c^	3.598**
	Flavor of garlic	2.17 ± 1.62^d^	4.72 ± 1.23^c^	5.94 ± 1.11^b^	6.78 ± 1.31^ab^	7.44 ± 1.46^a^	42.223***

All values are mean ± standard deviation (SD) (n = 25).

Different letters within the same row (a–d) differ significantly by Duncan’s multiple range test (P < 0.05).

**P < 0.01, ***P < 0.001.

In conclusion, total polyphenol and flavonoid contents increased proportionally to the amount of garlic powder added; as a result of the increased phenolic content, the DPPH and ABTS^+^ radical scavenging activities and reducing power increased. Water content did not vary across groups, whereas pH decreased with increasing amounts of garlic powder. The lightness of the fish cakes increased, whereas the redness and yellowness decreased with increasing amounts of garlic powder. All tested items of mechanical texture decreased with the increase in the added amount of garlic powder. The electronic tongue analysis showed increased intensities of saltiness, sourness, and umami as the amount of garlic powder increased. Across all garlic- powder groups, the electronic nose showed high peaks for volatile aromatic compounds as the amount of garlic powder increased, with the lowest trimethylamine peak—corresponding to reduced fishy odor—observed with the addition of 4% garlic powder. The preference score in the sensory evaluation was highest for the 3% garlic-powder group. Additionally, fishy odor decreased as the amount of garlic powder increased. The addition of garlic powder increased the antioxidant effects and umami flavor, consistent with the increase in various volatile aromatic compounds. The decrease in fishy odor had a major positive effect on the preference values. Therefore, the addition of garlic powder enhanced the preference and added functionality to the fish cakes. Our findings suggest that the addition of 3% garlic powder produces fish cakes with outstanding qualities.

## Data Availability

The data used to support the findings of this study are included within the article.
